# Coupling Interference between Eddy Current Sensors for the Radial Displacement Measurement of a Cylindrical Target

**DOI:** 10.3390/s22124375

**Published:** 2022-06-09

**Authors:** Weifeng Zhang, Jianguo Bu, Dongjie Li, Ke Zhang, Ming Zhou

**Affiliations:** 1 School of Aerospace Engineering, Tsinghua University, Beijing 100084, China; cheungwaiphen@163.com (W.Z.); drlee1994@126.com (D.L.); zmzlh@tsinghua.edu.cn (M.Z.); 2 Department of Military Vehicle Engineering, Military Transportation University, Tianjin 300161, China; bjg0073128@sina.com

**Keywords:** coupling interference, eddy current sensor, cylindrical target, finite element method, experiment

## Abstract

In the radial displacement measurement of a small-sized cylindrical target, coupling interference between eddy current sensors reduces the accuracy of the measurement. In this study, finite element method (FEM) simulation based on ANSYS Maxwell was adopted to investigate the relationships between the coupling coefficient of the sensors and different parameters including the lift-off, cylinder diameter, axis angle, material, and excitation frequency. The experimental results were consistent with the simulation results. The coupling interference between the sensors increased with the decrease in the lift-off and cylinder diameter. The coupling effect decreased significantly when the probe axis angle increased to 120°, and the decrease in the sensor sensitivity was acceptable. A polynomial fitting function fitted the output signal well. A compensation method was given based on the compensation necessity evaluation. The results showed that the error of the rotor motion track was significantly decreased after compensation.

## 1. Introduction

During the operation process of an eddy current sensor (ECS), the strength of the eddy current effect is influenced by the permeability, conductivity, shape, and distance of the measured target [1,2]. Based on these characteristics, different kinds of ECSs have been developed for different functions, such as defect detection [3,4], measurement of displacement [5,6], vibration [7], conductivity [8], and thickness [9]. Although ECSs have been widely applied in various industrial fields, further research has been devoted to the promotion of the accuracy and adaptability of sensors to satisfy higher requirements. Probes with different shapes, such as planar [10], rosette-like [11], butterfly-shaped [12], and flexible array [13,14] probes, have been developed to adapt to different situations. Planar coils allow increased sensitivity in the detection of micro-size defects, especially along the welded joints. Rosette-like and butterfly-shaped coils enable the detections of defects around bolt holes and screw threads with high sensitivity and low error rates. Flexible array sensors are used to inspect complex structures such as steel balls and welded structures, showing good sensitivity and adaptability to environmental variation. Multi-frequency [9,15] and pulsed [16] sensors are used to resist noise. Eddy current displacement sensors with self-temperature compensation have demonstrated ultra-stability [17,18]. The influence of heat treatment on the output of the sensor has been investigated based on analyses of the electromagnetic properties [19], and a new type of sensor independent of the sample electromagnetic properties was proposed [20]. The error produced by the tilt angle has been observed and eliminated by compensation [21].

Although all of these achievements have expanded the application fields of ECSs, there are still many limitations in the measurement of cylindrical targets. With the development of high-speed rotors, the clearances of bearings have become extremely narrow, and the maximum motion amplitude of a rotor can be less than 5 μm [22,23], bringing about much higher precision requirements in displacement measurements. In order to promote accuracy, the influences of the curvature, eccentricity, and interference should be considered. Ding et al. developed curved flexible coils for the measurement of gaps between curved surfaces and investigated their characteristics [24,25]. To eliminate the influence of the curvature on crack detection, Zhang et al. studied the lift-off noise of probe coils on curved specimens with small radii of curvature and presented a phase rotation and signal enhancement technique to eliminate the noise [26]. Zhan et al. studied the influence of eccentricity on cylindrical specimens and introduced a compensation method to reduce the error [27]. Sheng et al. studied the interference between arrayed ECSs [28]; however, the interference between sensors for radial displacement measurement has not been studied sufficiently. Consequently, during the operation of ECSs, the diameter of the cylindrical target is restricted to be much higher than the diameter of the sensing probe [29]. The minimum shaft diameter is 50.8 mm, and the recommended minimum diameter is 76.2 mm for Bently Nevada 3300 5 mm probes to reduce cross-talk interactions between probes. Researchers have sometimes had to employ other methods, such as the use of capacitance [30], laser [31], and electrostatic [32] devices, to measure the displacement and vibration of shafts.

Therefore, it is necessary to analyze the actual influence of the coupling interference between two ECSs for radial displacement measurement, based on which a compensation method could be given, and the application field of ECSs can be enlarged as a result. The regular pattern of the interference between the ECSs were investigated by finite element method (FEM) simulation analyses in this study. Experiments were employed to verify the simulation results and reveal the actual influence on the output signals. A compensation method based on application scenarios was presented to reduce the error.

## 2. Basic Principle

**Figure 1:**
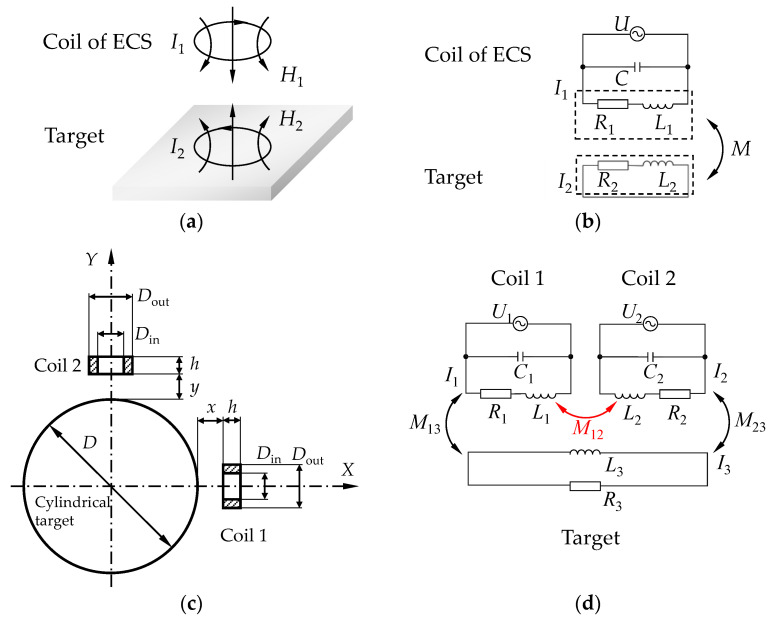
Operating principle and equivalent circuit models of eddy current sensors (ECSs) and detected targets. (**a**) Eddy effect on a detected target; (**b**) equivalent circuit model of an ECS and a target; (**c**) geometric graph of two ECSs located along radial directions of a cylindrical target; (**d**) equivalent circuit model of two sensors with mutual inductance and a target.

The operating principle of an ECS is based on Faraday’s law of electromagnetic induction. As shown in Figure 1a, when a coil is supplied with an alternating current (AC) *I*_1_, an alternating magnetic field *H*_1_ is generated, which induces an eddy current *I*_2_ in the detected target. The eddy current generates a magnetic field *H*_2_ in the opposite direction from *H*_1_. The principle of the eddy effect can be expressed by an equivalent circuit model, which is shown in Figure 1b.

The inherent resistance and inductance of the coil are *R*_1_ and *L*_1_, respectively. Similarly, the resistance and inductance of the conductor are *R*_2_ and *L*_2_, respectively. Mutual inductance between the coil and the conductor is *M*, which can be expressed as

(1) $$M=k\sqrt{L_{1}L_{2}},$$

where *k* represents the coupling coefficient. Based on Kirchhoff’s law, when the coil is driven by a current *I*_1_ with an angular frequency *ω*, the equivalent resistance *R*_s_ and inductance *L*_s_ of the coil can be derived as

(2) $$\left\{ \begin{array}{l} R_{s}=R_{1}+\frac{\omega^{2}M^{2}}{R_{2}^{2}+{\left(\omega L_{2}\right)}^{2}}R_{2}\\ L_{s}=L_{1}-\frac{\omega^{2}M^{2}}{R_{2}^{2}+{\left(\omega L_{2}\right)}^{2}}L_{2} \end{array}\right.$$

*M* can be influenced by the lift-off *x*; therefore, when the electrical conductivity *σ* and permeability *μ* of the conductor and the excitation frequency *f* of the coil are constant, where *f* = *ω*/2π, the equivalent impedance *Z*_s_ of the coil is a function of *x*. With a circuit transforming *Z*_s_ to an output signal *U*_out_, the displacement *x* is detected by measuring *U*_out_.

When several sensors operate simultaneously, the coupling interference should be taken into consideration. For a test system using two sensors, as shown in Figure 1c, the equivalent circuit is presented in Figure 1d. In this model, in addition to the mutual inductance between the coils and the detected object, which are expressed as *M*_13_ and *M*_23_, respectively, the mutual inductance of two coils, *M*_12_, adds extra impedance to the coils. Therefore, the coupling effect between two sensors can be represented by the values of *M*_12_ and *k*_12_.

Coil 1 and coil 2 are driven by currents *I*_1_ and *I*_2_, respectively, with an angular frequency *ω*, where *I*_1_ = *I*_2_. Supposing that the coils have equal mutual inductances (*M*_13_ = *M*_23_), according to Kirchhoff’s law, the equivalent resistance *R*_s1_ and inductance *L*_s1_ of coil 1 can be expressed as

(3) $$\left\{ \begin{array}{l} R_{s1}=R_{1}+\frac{2\omega^{2}M_{13}^{2}}{R_{3}^{2}+{\left(\omega L_{3}\right)}^{2}}R_{3}\\ L_{s1}=L_{1}-\frac{2\omega^{2}M_{13}^{2}}{R_{3}^{2}+{\left(\omega L_{3}\right)}^{2}}L_{3}+M_{12} \end{array}\right.$$

In contrast to Equation (2), the equivalent inductance of coil 1 is influenced by coil 2, because *M*_12_ contributes to the value of *L*_s1_. Therefore, it is important to discover the variation characteristics of *M*_12_ with certain parameters, by which the coupling interference between ECSs can be quantified.

## 3. Simulation Analyses

### 3.1. Theories of the Mutual Inductance of Two Coils

**Figure 2:**
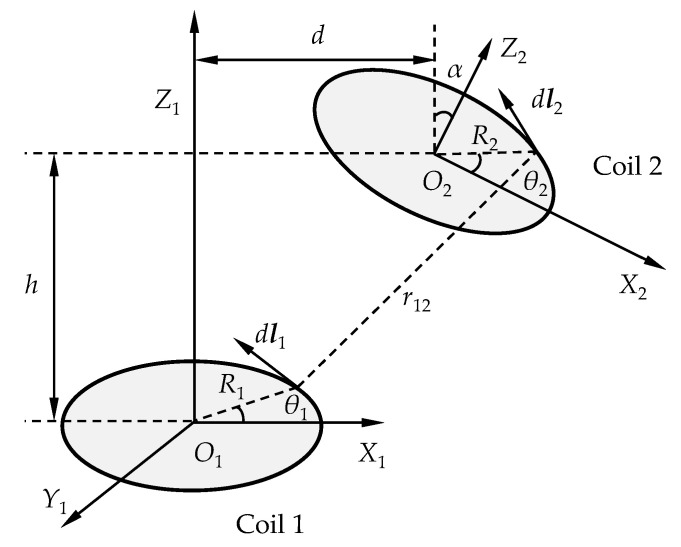
Configurations of two coils located at arbitrary positions.

For two coils located at arbitrary positions, as shown in Figure 2, the mutual inductance *M*_12_ of the coils can be calculated using Neumann’s equation [33]:

(4) $$M_{12}=\frac{N_{1}N_{2}\mu }{4\pi }\oint \oint \frac{1}{r_{12}}{dl}_{1}{dl}_{2}\;,$$

where *N*_1_, *N*_2_, *μ*, d***l***_1_, d***l***_2_, and *r*_12_ are the turns of coil 1, the turns of coil 2, the magnetic permeability of the medium in the space, the infinitesimal of the vector along the tangential direction of coil 1, the infinitesimal of the vector along the tangential direction of coil 2, and the distance between the two infinitesimals, respectively. Based on the geometrical relationships, Equation (4) can be expressed as

(5) $$M_{12}=\frac{N_{1}N_{2}\mu R_{1}R_{2}}{4\pi }\int_{0}^{2\pi }\int_{0}^{2\pi }\frac{\sin\theta_{1}\sin\theta_{2}\cos\alpha +\cos\theta_{1}\cos\theta_{2}}{r_{12}}d\theta_{1}d\theta_{2}$$

In Equation (5), *μ* = *μ*_r_*μ*_0_, where *μ*_r_ and *μ*_0_ are the relative permeability and the permeability of vacuum, respectively. *r*_12_ can be calculated by

(6) $$\begin{array}{l} r_{12}=[R_{1}^{2}+R_{2}^{2}+h_{}^{2}+d_{}^{2}+2dR_{2}\cos\theta_{2}\cos\alpha -2dR_{1}\cos\theta_{1}\\ -2R_{1}R_{2}(\cos\theta_{1}\cos\theta_{2}\cos\alpha +\sin\theta_{1}\sin\theta_{2})-2R_{2}h\cos\theta_{2}\sin\alpha {]}^{1/2} \end{array}$$

According to Equation (5), the mutual inductance *M*_12_ of coil 1 and coil 2 depends on the sizes and relative positions of the coils and the properties of the medium in the atmosphere. Therefore, for two coils with certain structures, as shown in Figure 1c, the lift-off values of the ECSs, which can be expressed as *x* and *y*, the diameter *D* of the cylindrical target, the axis angle *α* of the coils, and the permeability *μ* of the target have influences on the value of *M*_12_.

The simplified model described in Figure 2 is based on many assumptions. (1) The structures of the coil windings are equal in size and position. The assumption may lead to error when the coils are in large three-dimensional sizes. The model should be promoted, therefore, by considering the mutual inductance between each turn of the coils and combining them [34]. (2) The medium in the atmosphere is uniformly distributed. When the medium is composed of different materials, as shown in Figure 1c, the magnetic field is too complicated to be described by equations. (3) The eddy effect is not considered. The eddy current field generated in the target, which is relevant to the excitation frequency *f* of the coils and the conductivity *σ* of the target, as shown in Figure 1c, will affect the mutual inductance by changing the magnetic field. Therefore, the mutual inductance *M*_12_ and coupling coefficient *k*_12_ of the coils in Figure 1c should be described by the following equations:

(7) $$M_{12}=M\left(x,y,D,\alpha ,\sigma ,\mu ,f\right),$$

(8) $$k_{12}=k\left(x,y,D,\alpha ,\sigma ,\mu ,f\right)$$

The expressions of Equations (7) and (8) are too difficult to be obtained by theoretical calculations. Therefore, it is necessary for this study to discover the relationships between the parameters by FEM simulation and experimental analyses and construct fitting functions to describe the relationships.

### 3.2. FEM Simulation Modeling

**Figure 3:**
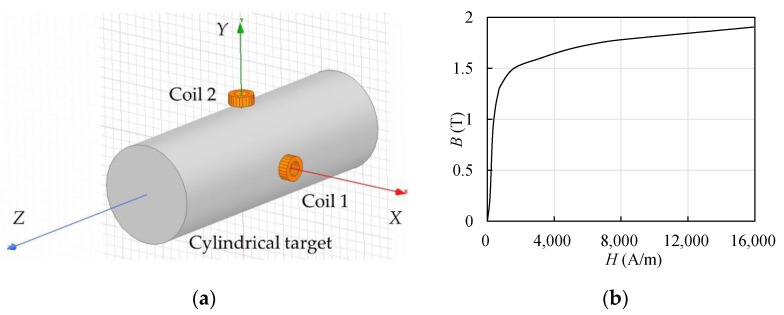
FEM simulation model established in ANSYS Maxwell. (**a**) Three-dimensional configuration of the simulation model; (**b**) *B*–*H* (magnetic flux density–magnetic field intensity) curve of steel 1008 in ANSYS Maxwell material library.

Theoretical solutions to actual eddy current fields and mutual inductances are excessively complicated; therefore, FEM simulation through software is optimal for most application scenarios because of its high accuracy, which has been proven by theoretical analyses [35] and experiments [36], and the low complexity of modeling. Two-dimensional (2D) models are recommended for axisymmetric objects [37,38] to reduce complexity and improve solving efficiency. As the locations of the ECSs for the radial displacement measurement of a cylindrical target change the axial symmetry, 2D models are no longer applicable. Therefore, three-dimensional (3D) simulation models were established in ANSYS Maxwell, which is a commonly used software [21,27,28], as shown in Figure 3a.

The materials of the coils, target, and atmosphere were set in the simulation model. The typical material of a coil is copper, whereas the materials of the detected conductors differ widely. Conductors are always classified as ferromagnetic and non-ferromagnetic materials according to their electromagnetic characteristics [39]. In this study, the properties of a ferromagnetic material, steel 1008, and a non-ferromagnetic material, aluminum, were both observed as detection targets. The main parameters of the above materials and the vacuum are listed in Table 1, which are included in the material library of ANSYS Maxwell. The magnetization process of a ferromagnetic material is nonlinear, resulting in the permeability being non-constant. Therefore, the permeability is reflected by the *B*–*H* curve, as shown in Figure 3b, where *B* is the magnetic flux density, and *H* is the magnetic field intensity. The coil is influenced by both the eddy effect and the magnetoresistance effect when detecting a ferromagnetic target, and the output is sensitive to the composition distribution [39]. The magnetization process of a ferromagnetic material is complex and unstable; therefore, the material of the detected target was aluminum unless noted otherwise. The atmosphere within the solution region was set as a vacuum.

To cover most ECSs in use, the excitation frequencies of the coils were set to 1, 10, 100, and 1,000 kHz. The excitation frequency of a coil has a significant influence on the penetration depth *δ* of the eddy current. The relationship is described by Equation (9).

(9) $$\delta =\frac{1}{\sqrt{\pi f\mu \sigma }}$$

The outer diameter *D*_out_, inner diameter *D*_in_, and height *h*, of each coil were 5, 3, and 2 mm, respectively, as shown in Figure 1c. The diameter of the coil is represented by the outer diameter; thus, *D*_coil_ = *D*_out_. 

Different cylinder diameters *D* were given to analyze the influence of the cylinder diameter on the coupling effect. The length of the cylindrical target *L* was set to 12 times *D*_coil_, aiming to reduce boundary errors. Coils 1 and 2 were located along *X* and *Y* directions, respectively. The lift-off values were *x* and *y*, respectively, and were adjustable.

The meshing operation of the coils was set as “inside selection” and “length based,” where the element sizes were limited to within 0.5 mm. Regarding the skin effect of the eddy current, the meshing operation of the cylindrical target was set as “on selection” and “skin depth based.” The penetration depth was calculated based on Equation (9), and eight layers of elements were given on the skin to ensure sufficient accuracy. The maximum element sizes of the cylindrical target were limited to 3 mm. Moreover, the elements of the surface corresponding to the coils, where the eddy current was mainly generated, were refined. The solution region was set to “300%” in each direction. The solution type was set as “eddy current,” and skin effects were considered.

### 3.3. Influence of Lift-Off on Coupling Effect

As discussed in Section 3.1, for two coils in a vacuum environment, the relative positions have a significant influence on their mutual inductance. As for the ECSs in a cylindrical target displacement measurement, the directions of the two sensors are always vertical. The primary variable is the lift-off, representing the gap between the sensor and the target. The lift-off of coil 1 was kept constant (*x* = 1 mm). The lift-off of coil 2 varied from 50 mm to 0.5 mm. When the lift-off was sufficiently large (*y* > 50 mm), the influence could be ignored. The diameter of the cylindrical target was 20 mm, which was four times the diameter of the coil.

**Figure 4:**
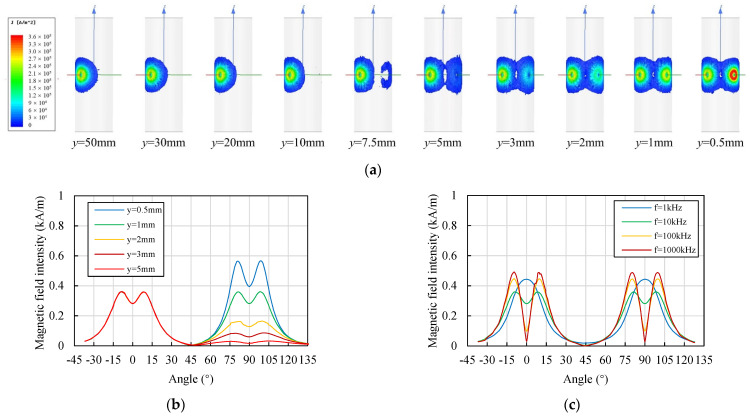
Change in the eddy current density and magnetic field intensity distributions of the target with different lift-off values and excitation frequencies of coil 2 (material of the target: aluminum, *x* = 1 mm, *D* = 20 mm, phase of excitation current: 0°). (**a**) Change in the eddy current density distribution on the target with different lift-off values (*f* = 10 kHz); (**b**) change in the magnetic field intensity distribution along the circumferential direction with different lift-off values of coil 2 (*f* = 10 kHz); (**c**) change in the magnetic field intensity along the circumferential direction with different excitation frequencies of the coils (*y* = 1 mm).

Figure 4a shows the variations in the distribution of the eddy current field with the lift-off of coil 2. When *y* > 10 mm, the eddy current generated by coil 2 was weak. With the decrease in *y* from 10 mm to 0.5 mm, the eddy current generated by coil 2 increased significantly. The increasing eddy current field covered an overlapping area with the field generated by coil 1. Figure 4b shows the change in the magnetic field intensity *H* along the circumferential direction with different values of the lift-off *y*, where the angle was measured from the *X* axis along the counterclockwise direction. *H* decreased with the increase in *y*. Figure 4c shows the change in *H* with different values of the excitation frequency *f*. With the increase in *f*, *H* was more concentrated at the surface area of the target facing the coils.

**Figure 5:**
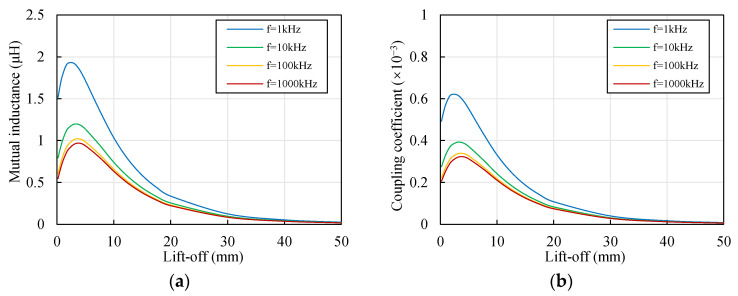
Change in the coupling effect of the coils with the lift-off of coil 2 (material of target: aluminum, *x* = 1 mm, *D* = 20 mm). (**a**) Change in the mutual inductance of the coils with the lift-off of coil 2; (**b**) change in the coupling coefficient of the coils with the lift-off of coil 2.

Figure 5 shows the changes in the mutual inductance *M*_12_ and the coupling coefficient *k*_12_ with the lift-off *y* and the excitation frequency *f*. The trend in *k*_12_ was consistent with the trend in *M*_12_. *k*_12_ was dimensionless; therefore, this paper mainly discusses the change in *k*_12_. As shown in Figure 5b, different curves had similar trends, *k*_12_ reached the peak when *y* was around 3 mm. With the decrease in the lift-off, the distance between the two coils decreased and the eddy current density generated by coil 2 increased. The changes might have resulted in the increase in the coupling coefficient. The magnetic field intensity was more concentrated when *y* was small, as shown in Figure 4b. This may have resulted in the decrease in the coupling coefficient when *y* was less than 3 mm. *k*_12_ decreased with the increase in *f*, which corresponded well with the trend in the two parallel sensors [28]. The excitation frequency influenced the distribution of the induced magnetic field. A higher frequency resulted in a stronger skin effect, and the generated magnetic field became more concentrated, as shown in Figure 4c.

Therefore, increasing the excitation frequency of the ECS was beneficial for reducing interference for the cylindrical aluminum target. The promotion became weak when the excitation frequency exceeded 100 kHz, however. Proper lift-off of the ECS has the potential to reduce interference, and it is recommended to keep the lift-off as far as possible from the value where the coupling coefficient reaches its peak.

### 3.4. Influence of Cylinder Diameter on the Coupling Effect

ECSs are widely applied in cylindrical target displacement measurements. The cylinder diameter determines the distance between two sensors and influences the coupling effect. The diameter of a cylindrical target varies significantly; therefore, it is necessary to discuss its influence on the radial sensors. The lift-off values of the two coils were set to typical values (*x* = *y* = 1 mm).

**Figure 6:**
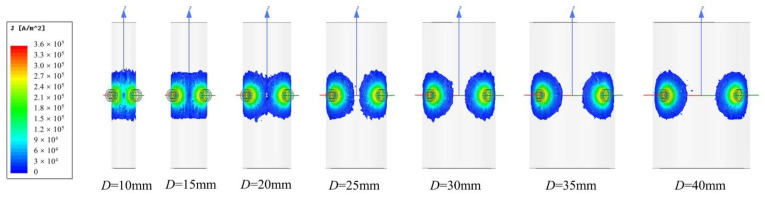
Change in the eddy current distribution on the targets with different cylinder diameters (material of the target: aluminum, *x* = *y* = 1 mm, *f* = 10 kHz).

Figure 6 shows the variations in the distribution of the eddy current field with the change in the cylinder diameter *D*. When *D* > 20 mm, the eddy current fields generated by the two coils retained enough distance to avoid strong interference. With the decrease in *D* from 20 to 10 mm, the overlapping area of the two eddy current fields increased significantly, which may have resulted in an increase in the mutual interference.

**Figure 7:**
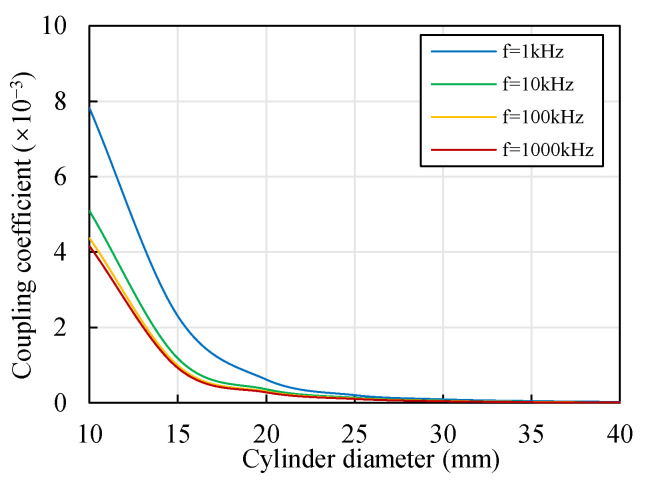
Change in the coupling coefficient of the coils with the diameter of the target (material of the target: aluminum, *x* = *y* = 1 mm).

Figure 7 shows the change in the coupling coefficient *k*_12_ with the cylinder diameter *D* and excitation frequency *f*. *k*_12_ decreased with the increase in *f*, which corresponded well with Figure 5b. Moreover, *k*_12_ decreased with the increase in *D*. There may have been two reasons for this phenomenon. On the one hand, with the decrease in the cylinder diameter, the distance between the two coils decreased accordingly, resulting in an increase in the coupling coefficient. On the other hand, the shape of the cylindrical target influenced the eddy current distribution, as shown in Figure 6, which resulted in a change in the coupling coefficient.

**Figure 8:**
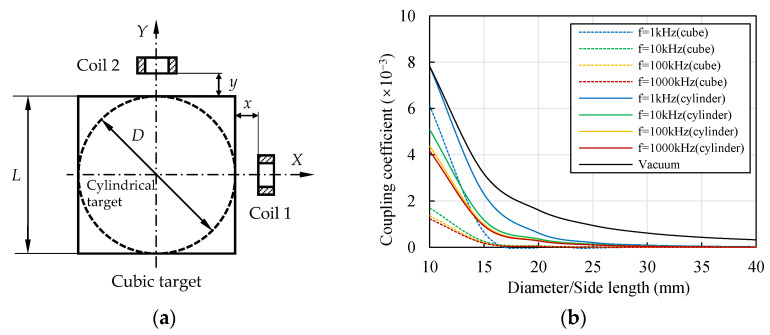
Contrast of the coupling coefficients for cylindrical and cubic targets (material of the targets: aluminum, *x* = *y* = 1 mm). (**a**) Model of the cubic and cylindrical targets; (**b**) change in the coupling coefficient of the coils with the sizes of the targets.

In order to verify the shape effect described above and distinguish it from the effect of the distance, an additional model was established to contrast the coupling effects with the same distance and different shapes. A cubic target was considered, as shown in Figure 8a, where the side length of the square section equaled the diameter of the cylindrical target (*L* = *D*). The coils had the same relative positions when the cylindrical target was replaced by the cubic target, although the shape of the target had changed. Moreover, the coupling coefficient of the two coils in a vacuum was calculated.

The results are compared in Figure 8b. The coupling coefficient did not change with the excitation frequency in a vacuum. The existence of both a cylindrical target and a cubic target reduced the coupling coefficient. The variation in the coupling coefficient with the side length of the square section was similar to the change with the cylinder diameter. With the decrease in the side length, the coupling coefficient significantly increased. Compared with the cylindrical target with an equal size, the coupling coefficient of the cubic target was smaller. A possible reason for this was that the overlapping area of the two eddy current fields was smaller when the cylindrical target was replaced by the cubic target. These results provide support to the shape effect on the eddy current distribution mentioned above, the shape of the target influenced the coupling coefficient, even if the relative positions of the two coils were unchanged. 

Therefore, the shape of the target had an influence on the coupling effect. The circular section resulted in a higher coupling coefficient than the square section. The dominant influence element was the relative position, however. When ECSs are applied for the radial displacement measurement of a cylindrical target with a small diameter, coupling effects cannot be neglected and compensation is necessary. The threshold for neglecting the coupling effect depends on the accuracy requirement. Higher accuracy requires a higher *D*/*D*_coil_ ratio.

### 3.5. Influence of Coil Axis Angle on the Coupling Effect

**Figure 9:**
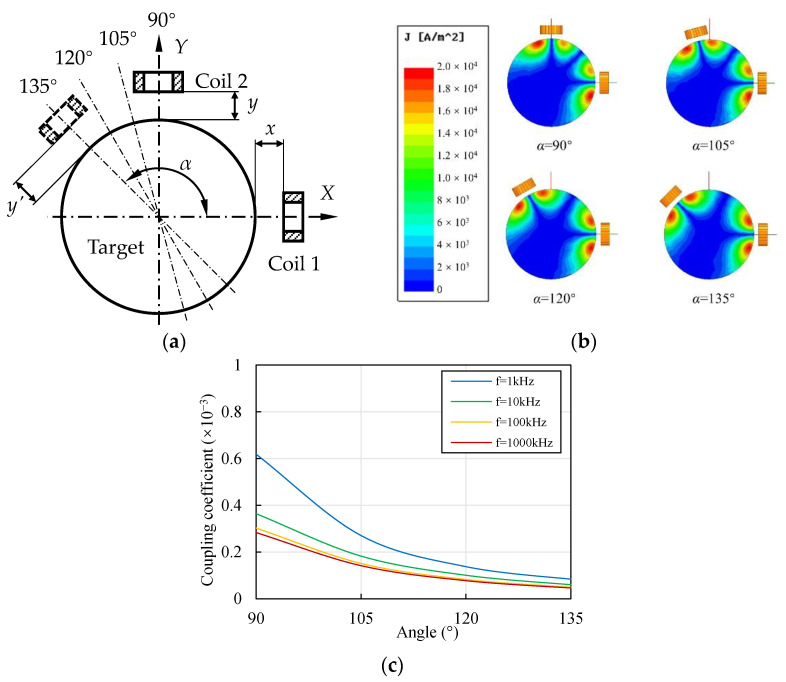
Contrast of coupling effects with different axis angles. (**a**) Geometric graph of two ECSs along radial directions with different coil axis angles. (**b**) change in the eddy current distribution of the target with the axis angle (material of the target: aluminum, *D* = 20 mm, *x* = *y* = 1 mm, *f* = 1 kHz); (**c**) change in the coupling coefficient with axis angle (material of the target: aluminum, *D* = 20 mm, *x* = *y* = 1 mm).

Typically, the angle between coil axes is 90°. A larger axis angle might reduce the coupling effect, however, because the distance between the coils increases with the increase in the axis angle. Moreover, the overlapping area decreases with the increase in the axis angle. Both changes show possibilities for reducing the coupling effect. A series of angles, from 90° to 135° with steps of 15°, was specified in the model to evaluate their influence, as shown in Figure 9a. If the angle exceeded 135°, the sensitivity of the sensor output was as low as around 1/$\sqrt{2}$ ≈ 0.707 of the original output, which is always unacceptable. Therefore, the angle was controlled to within 135°.

Figure 9b shows the variations in the eddy current field distribution with the coil axis angle *α*. With the increase in *α* from 90° to 135°, the overlapping area of the two eddy current fields decreased significantly, which could have resulted in a decrease in the mutual interference. Figure 9c shows the change in the coupling coefficient with the axis angle. With the increase in *α* from 90° to 135°, *k*_12_ decreased significantly, which was consistent with the variation behavior of the eddy current distribution shown in Figure 9b. For *f* = 1 kHz, *k*_12_ decreased to around 44%, 22%, and 14% when α increased from 90° to 105°, 120°, and 135°, respectively. When *f* > 10 kHz, *k*_12_ decreased to around 50%, 27%, and 16% when *α* increased from 90° to 105°, 120°, and 135°, respectively.

Therefore, the angle between the coil axes had a significant influence on the coupling effect. Increasing the angle was beneficial for reducing the mutual interference. When *D*/*D*_coil_ = 4, the angle is recommended to be 120° to reduce the coupling coefficient to about 27%. If the sensor is not located along the vertical direction, the output *y*′ should be transformed to *y* through the following equation based on their geometric relationship:

(10) $$\Delta y=\frac{\Delta y^{\prime }}{\cos\left(\alpha -90^{\circ }\right)}+\Delta x\tan\left(\alpha -90^{\circ }\right)$$

The sensitivity of *y*′ is around $\sqrt{3}$/2 ≈ 0.866 of *y* when *α* = 120°, which is acceptable in most situations.

### 3.6. Influence of Material on Coupling Effect

Aluminum is mainly applied in non-rotational structures, while rotational structures primarily adopt steel materials to obtain better comprehensive performances. Due to the significant differences in the conductivity and permeability of these materials, which are both essential to the eddy current field distribution, cylindrical targets of different materials might have significantly different coupling effects. Thus, aluminum and steel cylindrical targets were compared with a vacuum environment. Steel 1008 was selected as typical steel. The parameters of these materials are shown in Table 1 and Figure 3b.

**Figure 10:**
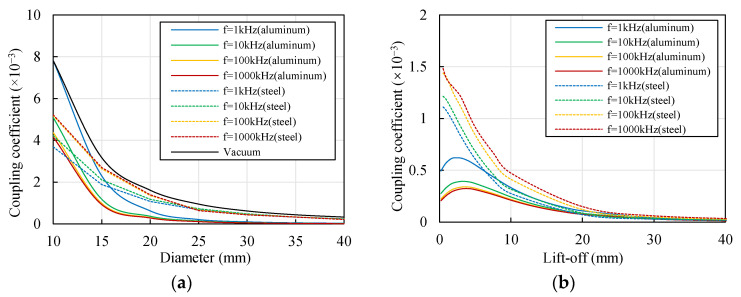
Change in the coupling coefficient of aluminum and steel cylindrical targets. (**a**) With the cylinder diameter (*x* = *y* = 1 mm); (**b**) with the lift-off (*x* = 1 mm, *D* = 20 mm).

Figure 10a shows the change in the coupling coefficient with the cylinder diameter using different materials. Compared with the coils in a vacuum, the existence of a metal cylindrical target reduced the coupling coefficient of the coils. *k*_12_ decreased with the increase in the diameter of the steel cylindrical target, which was similar to the trend in the aluminum cylindrical target. The descending slope was much smaller, however. There was difference between the two materials in terms of the change with the excitation frequency. When the cylinder diameter *D* < 20 mm, the coupling coefficient *k*_12_ of the steel material increased with the increase in the excitation frequency *f*. When *D* > 25 mm, *k*_12_ did not change significantly with the change in *f*. Moreover, the *k*_12_ curves showed little difference at 100 and 1,000 kHz. As shown in Figure 10b, the variation characteristics of the coupling coefficient *k*_12_ with the lift-off *y* of coil 2 when using steel material was different from those when using aluminum. The coupling coefficient of the steel material decreased with the increase in the lift-off in the whole range, while the coupling coefficient of the aluminum material reached the peak when *y* was around 3 mm.

Therefore, the coupling effect had much different variation characteristics when different materials were adopted. Different materials required different excitation frequencies to reduce the interference. Moreover, the coupling coefficient can be significantly reduced by increasing the diameter of the aluminum cylindrical target to 4–5 times the coil diameter, while the method was not appropriate for the steel target. This indicated that compensation is commonly necessary for steel material to obtain high accuracy. When detecting an aluminum material, the coupling coefficient can be reduced by both increasing and reducing the lift-off, while the coupling coefficient can only be reduced by increasing the lift-off when detecting a steel material.

## 4. Experiments and Discussions

As determined via the FEM simulation, the lift-off, cylinder diameter, axis angle, and material were essential for the coupling effect of the ECSs for the radial displacement measurement of a cylindrical target, which was revealed by the changes in the coupling coefficient. The variation characteristics are the bases of sensor design and selection. In order to eliminate the errors, the actual influence on the output signal should be observed.

### 4.1. Experiment Design and Instruments

**Figure 11:**
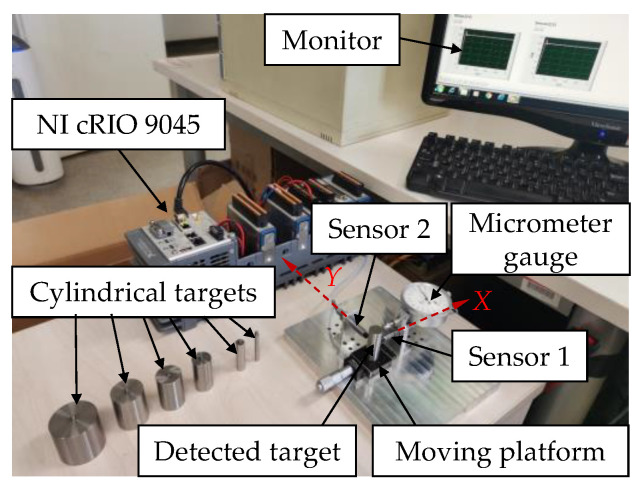
Experimental setup for the measurement of the coupling effect of the sensors.

As shown in Figure 11, a test platform was developed by the authors to measure the signal outputs of two sensors located along *X* and *Y* directions. A cylindrical target was placed on a moving platform. The lift-off was measured by a micrometer gauge. Probes of the sensors were placed along the *X* and *Y* directions of a cross section at a certain height. Probes were fixed on two supports, which could be moved on *X* and *Y* directions. Their displacements were measured by scales. Additional tools were used to fix the probes with certain deflection angles.

The ECS type was ZA210503, whose measurement range was 1 mm. The diameter of the probe was 5 mm. The sensors were calibrated on 45# steel before leaving the factory. The sensitivity was around 10 V/mm. They needed to be recalibrated for this study, as both the materials and the shapes of the targets were different.

A series of cylindrical targets were tested. The diameters of the cylindrical targets covered 5 mm to 60 mm, corresponding to target/coil diameter ratios from 1 to 12. As steel materials are more applied in rotors and the coupling effects of steel materials are more evident than those of aluminum materials, an ultra-high-strength steel, 42CrMo, was selected. The material is widely used in high-speed rotors, such as in turbochargers [40,41]. The cylindrical targets underwent heat treatment to reach the hardness of HRC 28–32. The surface roughness (Ra) was controlled to within 0.4 to reduce the error. The 7075 aviation aluminum-alloy was selected for comparison, which is commonly applied in applications requiring high strength.

An NI compact RIO 9045 test system with an NI 9215 analog input module was used to collect the output signals of the ECSs. The NI 9215 included four simultaneously sampled analog input channels with a 16-bit analog-to-digital converter (ADC) resolution. The sample rate was 100 kS/s per channel. LabVIEW was used to condition the signals with filters and show the stable results.

### 4.2. Calibration of Eddy Current Sensors on Cylindrical Targets

The relationship between the lift-off and the output signal changes with the shape and material of the detected target. Moreover, because heat treatment has a significant influence on the electromagnetic parameters, the influence of the material processing method should be considered. Therefore, it is necessary to recalibrate the sensors and the change in the output signals can be transformed more accurately to the change in the lift-off values to evaluate the actual error.

**Figure 12:**
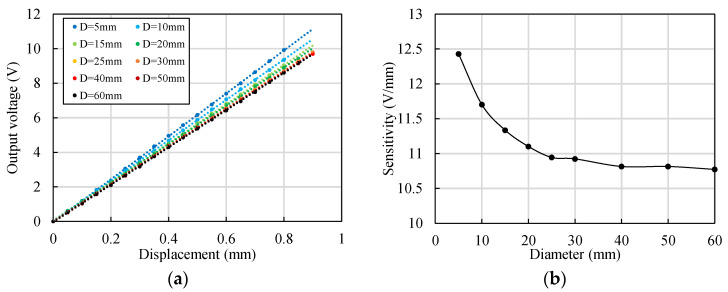
Output characteristics of the ECS when detecting cylindrical targets with different diameters (material of the target: 42CrMo steel). (**a**) Change in the output voltage with the displacement; (**b**) change in the sensitivity with the diameter.

The characteristics of the output voltage *U* of the ECS when detecting cylindrical targets with diameters ranging from 5 mm to 60 mm were tested. The results are shown in Figure 12a. The linearity of each measurement was acceptable. The slope of the *U*–*x* curve represented the sensitivity of the sensor, which is shown in Figure 12b. The increase in the sensitivity resulted in a decrease in the measurement range. The sensitivity decreased with the increase in the diameter and remained nearly unchanged when the diameter exceeded 40 mm.

With the same method, the sensitivity of the ECS when detecting a cylindrical aluminum alloy target was tested. The sensitivity was 18.6 V/mm for a cylindrical target with a diameter of 20 mm. Although ferromagnetic materials have significantly different eddy effects from non-ferromagnetic materials, the primary difference of the output signal is that the sensitivity for ferromagnetic materials is commonly smaller.

### 4.3. Influence of Coupling Effect on Measured Output Signal

**Figure 13:**
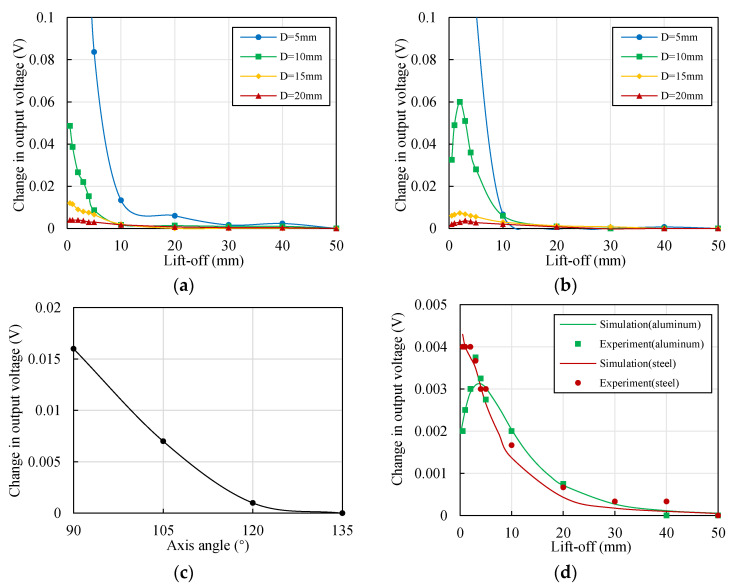
Change in the output voltage *U*_1_: (**a**) With the lift-off of sensor 2 (material of the target: 42CrMo steel, *x* = 1 mm); (**b**) with the lift-off of sensor 2 (material of the target: 7075 aluminum alloy, *x* = 1 mm); (**c**) with the axis angle (material of the target: 42CrMo steel, *x* = *y*’ = 1 mm, *D* = 15 mm); (**d**) comparison of the experimental and simulation results (*x* = 1 mm).

The output signal *U*_1_ of sensor 1, which was located along the *X* direction, was measured with and without sensor 2, which was located along the *Y* direction. The lift-off of sensor 2 was adjusted from around 0 to 50 mm. *U*_1_ decreased when sensor 2 was present, and the absolute values of the changes for the steel and aluminum materials are shown in Figure 13a,b, respectively. The results in both figures are consistent with the simulation results, as shown in Figure 10b. The change in *U*_1_ (|Δ*U*_1_|) was very high when *D* was as small as 5 mm. For the 42CrMo steel material, |Δ*U*_1_*|* decreased with the increase in *y*. For *D* > 10 mm, |Δ*U*_1_*|* could be ignored when *y* exceeded 10 mm. |Δ*U*_1_*|* reached about 0.05 V, representing a displacement shift of about 4 μm, when *D* = 10 mm and *y* = 0.5 mm. For the 7075 aluminum alloy material, |Δ*U*_1_*|* reached the peak when *y* was around 2 mm. The sensitivity of the 7075 aluminum alloy was much higher than the sensitivity of the 42CrMo steel; therefore, an equal |Δ*U*_1_*|* determined a smaller displacement for the 7075 aluminum alloy than that of the 42CrMo steel.

Another experiment was performed to verify the actual influence of the axis angle on the output voltage. The diameter of the cylindrical target was 15 mm. The position of sensor 1 was unchanged (*x* = 1 mm, *α* = 0°). Sensor 2 was placed at positions with different axis angles, from 90° to 135°, and the lift-off was unchanged (*y*’ = 1 mm). The results are shown in Figure 13c. |Δ*U*_1_*|* decreased with the increase in *α*. The value reached 50% when *α* = 105° and 10% when *α* = 120°. |Δ*U*_1_*|* was very small when *α* = 135°. The trend was consistent with the simulation results, as shown in Figure 9c.

Further experiments were conducted, proving that the interference brought by sensor 2 had little influence on the sensitivity of sensor 1.

The measurement of |Δ*U*_1_*|* at full scale is complex. However, the trend in the simulation fitted well with the experimental results. Thus, it is possible to transfer the simulation results to the output voltage by measuring only one single point (*y* = 1 mm, for example). The simulated coupling coefficient and the measured |Δ*U*_1_| when *y* = 1 determined a scale coefficient, and the coupling coefficients multiplied by the scale coefficient were |Δ*U*_1_| at full scale, as shown in Figure 13d. This proved that the simulation results fitted well with the experimental results for both the aluminum and steel materials.

### 4.4. Compensation Method

**Figure 14:**
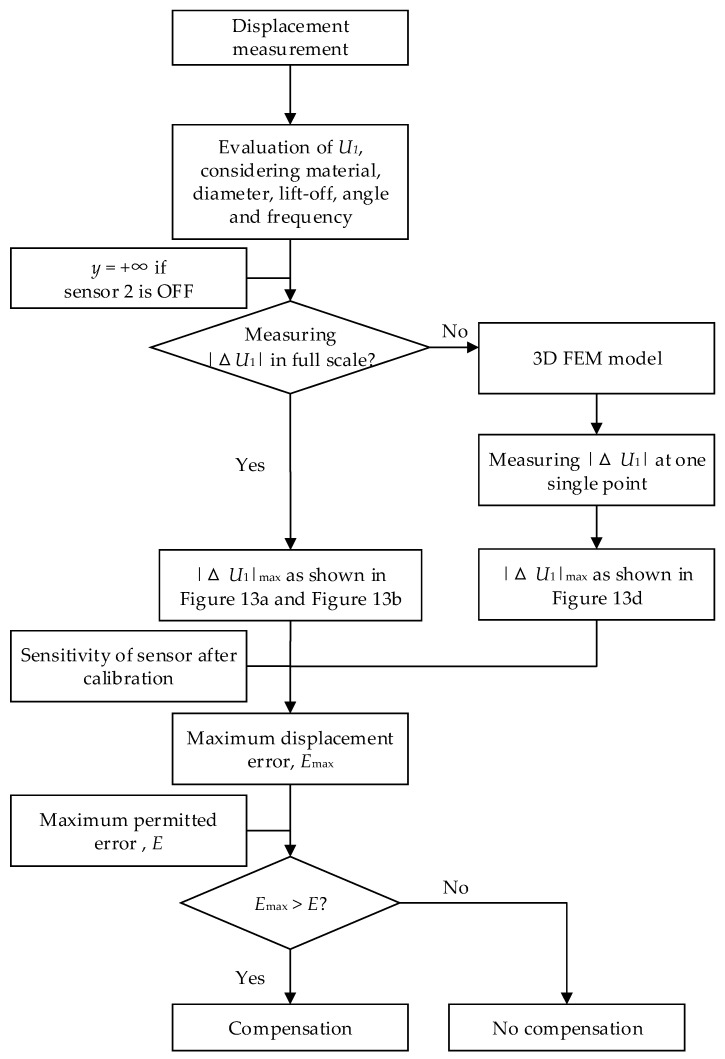
Flow diagram for the necessity estimation of compensation.

The changes in the output voltage caused by the coupling interference have many influencing factors; therefore, the coupling effect might be neglected in some application scenarios, such as when the diameter of an aluminum target exceeds eight times the diameter of the ECS coil. This paper proposes a method to estimate the necessity of performing compensation. The flowchart is shown in Figure 14, and the primary processes are as follows.

1. The maximum value of |Δ*U*_1_*|* is evaluated. The application scenario is certain; thus, parameters including the material and diameter of the target, the diameter, axis angle, and excitation frequency of the ECS coils are constant. The lift-off values of sensors are typically variable. Therefore, with the change in lift-off *y* in a certain range, where *y* = +∞ if sensor 2 is OFF, the variations in |Δ*U*_1_*|* should be estimated. Two methods, either by experiments or FEM simulation, can be adopted. The values of |Δ*U*_1_*|* could be measured at full scale, and the maximum value of |Δ*U*_1_*|*, marked as |Δ*U*_1_*|*_max_, can be obtained from the experimental results, as shown in Figure 13a,b. The values of |Δ*U*_1_*|* can also be calculated by obtaining the values of the coupling coefficient in a 3D FEM simulation model, measuring the value of |Δ*U*_1_| at one single point, and transforming the values of coupling coefficient to |Δ*U*_1_| at full scale. Then, the value of |Δ*U*_1_*|*_max_ can be obtained from the results, as shown in Figure 13d.

2. |Δ*U*_1_*|*_max_ is transformed to the maximum value of displacement error *E*_max_. According to the calibration data shown in Figure 12a,b, the maximum value of displacement error, *E*_max_, can be obtained by dividing |Δ*U*_1_*|*_max_ by the sensitivity of the ECS.
3. *E*_max_ is contrasted with the value of the maximum permitted displacement error *E*. In the measurement of displacement, the maximum permitted error is always given. The permitted error is typically within 1 μm, for example, for a rotor with a bearing clearance of 10 μm, where the corresponding eccentricity error is 0.1. If the maximum value of displacement error *E*_max_ exceeds the permitted value *E*, compensation should be employed.

**Figure 15:**
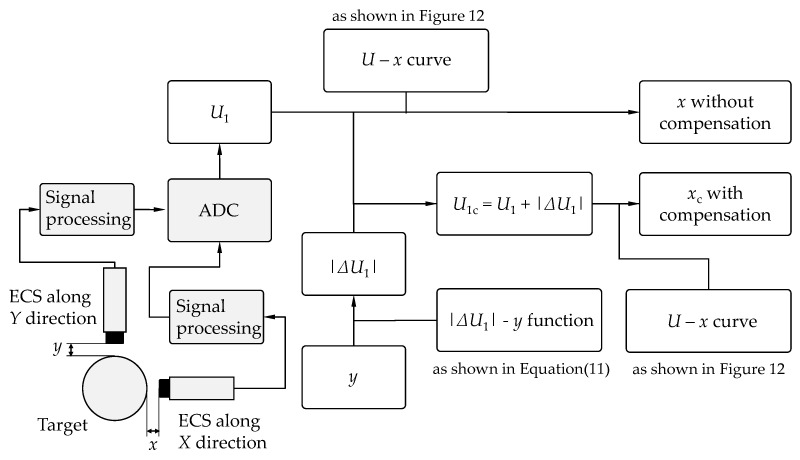
Flow diagram for the output voltage compensation of ECS.

Based on the discussions above, the compensation should be employed, if necessary, according to the flowchart shown in Figure 15. The primary processes are as follows.

(1) The output voltages of the eddy current sensors are collected. Signal processing circuits are necessary, which can provide excitation voltages to the coils, transform the values of impedance to voltages, and process the signals by amplifiers and filters. Then, the output voltage *U*_1_ of sensor 1 along the *X* direction is collected by an ADC module. The resolution of displacement measurement depends on the resolution of the ADC module; therefore, an ADC module with at least 12-bit resolution is recommended.
(2) A compensation voltage is added to the original output voltage. The value of *U*_1_ contains the coupling interference of sensor 2 along the *Y* direction, which results in a decrease in voltage by |Δ*U*_1_*|*; therefore, the value of |Δ*U*_1_*|* should be compensated to *U*_1_. The value of |Δ*U*_1_| depends on the lift-off value *y* of sensor 2. Typically, *y* is transformed to |Δ*U*_1_| by a |Δ*U*_1_|–*y* function based on polynomial fitting, which is subsequently discussed in detail. The value of *y* can be obtained directly by sensor 2 if the lift-off is within the measurement range. Otherwise, *y* should be measured by other methods. The output voltage with compensation is *U*_1c_, where *U*_1c_ = *U*_1_ + |Δ*U*_1_*|.*
(3) The output voltage with compensation *U*_1c_ is transformed to the displacement *x*_c_. The value of the displacement can be calculated based on the value of output voltage and the sensitivity obtained in Figure 12.

In order to calculate the value of |Δ*U*_1_| by *y* in a continuous range, there are two methods, linear interpolation and polynomial fitting, which are mainly used by researchers. Linear interpolation is commonly used to express unknown functions in a continuous range based on the statistics of limited quantities. The requirement for the accuracy of the statistics is high. It is preferred in fields where the function is hard to express directly. Therefore, polynomial fitting is more commonly adopted in most fields.

**Figure 16:**
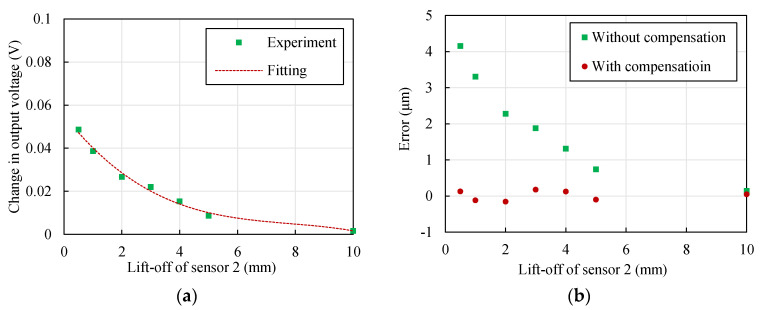
Compensation results using the polynomial fitting method (material of the target: 42CrMo steel, *D* = 10 mm, *x* = 1 mm). (**a**) Contrast of the polynomial fitting results and experimental results; (**b**) contrast of the displacement measurement errors with and without compensation.

The function |Δ*U*_1_|–*y* when *D* = 10 for the steel material was obtained through the polynomial fitting method. |Δ*U*_1_| was very low when *y* exceeded 10 mm; therefore, the range of *y* was [0.5,10]. The fitting function is shown as follows:

(11) $$\left|\Delta U_{1}\right|=k_{3}y^{3}+k_{2}y^{2}+k_{1}y+k_{0},$$

where the coefficients *k_i_* (*i* = 0,1,2,3) were obtained by the least squares method. The values were as follows: *k*_0_ = 5.52 × 10^−2^, *k*_1_ = −1.71 × 10^−2^, *k*_2_ = 2.04 × 10^−3^, *k*_3_ = −8.71 × 10^−5^. The units of |Δ*U*_1_| and *y* were V and mm, respectively. A comparison of the experimental results and fitting function is shown in Figure 16a. The fitting function fitted well with the experimental results. The corresponding |Δ*U*_1_| could be obtained by substituting *y* into Equation (11). 

Figure 16b shows the error with and without compensation. The displacement error was reduced to within ±0.2 μm after compensation, whereas the maximum error was more than 4 μm before compensation. Therefore, the displacement measurement could be significantly promoted by polynomial fitting compensation.

### 4.5. Motion Tracks of a Cylindrical Target with and without Compensation

**Figure 17:**
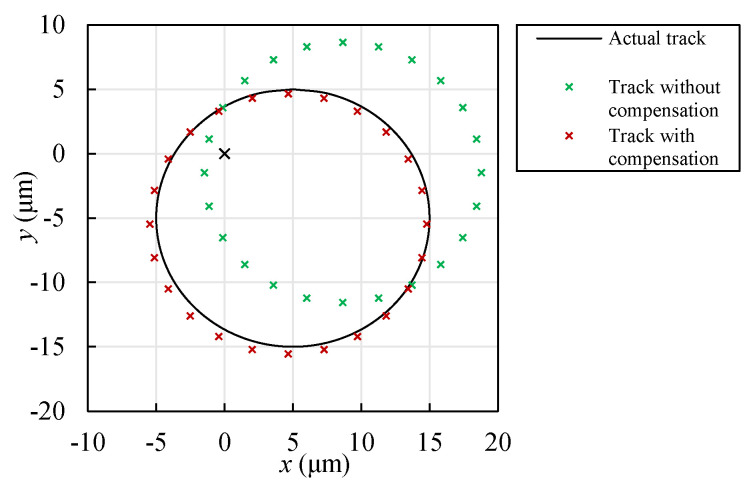
Contrast of the rotor tracks with and without compensation.

In order to demonstrate the effect of the compensation, a simulation was performed to compare the motion tracks of a rotor center with and without compensation. For a rotor supported by aero-foil bearings, because the surface of a foil bearing is soft, it is difficult to detect the geometric center of the bearing. Therefore, the measured track of the rotor center is typically based on relative positions, and the absolute positions are commonly uncertain [42]. One possible method to obtain the absolute positions of the rotor center is to measure the absolute displacement of the shaft to the ECSs. For a rotor with a diameter of 10 mm and a motion track, as shown in Figure 17, its output voltages were measured. Two methods were taken to transfer the voltages to motion tracks. The traditional method is to transfer the voltage based on the calibration data, as shown in Figure 12, without compensation. This paper proposes a new method by adding a compensation voltage to the measured voltage, as shown in Figure 15. The results in contrast with the actual track are shown in Figure 17. The motion track without compensation had an error of around 5 μm because the coupling effect decreased the output voltages of both sensors. The error was less than 1 μm after compensation, which was a result of the nonlinearity of the sensor output and the fitting error of the polynomial fitting function, proving that the compensation method was effective.

## 5. Conclusions

Coupling interference of ECSs for the radial displacement measurement of a cylindrical target was analyzed by FEM simulation, and different influencing factors and their contributions to the changes in the coupling coefficient have been discussed. Experiments were carried out to determine the actual influence of important factors on the output signals, and a compensation method was introduced. The primary conclusions are as follows:

(1) The simulation and experimental results agreed closely, proving that the lift-off, cylinder diameter, axis angle, material, and excitation frequency influenced the coupling effect between the sensors. The coupling coefficient decreased with the increase in the lift-off, cylinder diameter, and axis angle. For different materials, the coupling coefficient changed differently with frequency.
(2) Coupling interference decreased the output voltage of an ECS. The necessity of compensation should be estimated by considering the permitted displacement error and the maximum error generated by coupling interference. If compensation is necessary, compensation voltage can be obtained by polynomial fitting.
(3) The compensation method can be used to measure the absolute position of a rotor, and the effect was proven with a simulation model, showing that the error significantly decreased.

This paper provides a method to quantify the coupling effect between two ECSs for the radial displacement measurement of a cylindrical target. The compensation enables better accuracy of the motion track measurement of a rotation shaft or the position measurement of a cylindrical specimen. Moreover, errors due to coupling interference can be eliminated by changing the parameters of the measurement system, including the axis angle and excitation frequency of the coils, and the material and size of the target. 

The compensation method shows potential in the coupling effect compensation of any sensors located within close ranges, such as at two nearby surfaces of a cubic target. Further research could be concentrated on the study of the coupling effect of two or more sensors with coils in different shapes in the detection of various targets.

## 6. Patents

Zhang, W.; Li, D.; Xie, Z.; Huang, X.; Lan, X.; Zhang, K.; Zhou, M. Calibration device and method of eddy current sensor for rotating shaft displacement measurement. CN113251909B, 11 March 2022.

## Figures and Tables

**Table 1:** Electromagnetic parameters of the materials included in the simulation model.

Material	*σ* (MS/m)	*μ*_r_
Vacuum	0	1
Copper	58	0.999991
Aluminum	38	1.000021
Steel 1008	2	referring to the *B*–*H* curve shown in Figure 3b

## Data Availability

Not applicable.

## References

[1] Garcia-Martin J., Gomez-Gil J., Vazquez-Sanchez E. (2011). Non-destructive techniques based on eddy current testing. Sensors.

[2] AbdAlla A.N., Faraj M.A., Samsuri F., Rifai D., Ali K., Al-Douri Y. (2018). Challenges in improving the performance of eddy current testing: Review. Meas. Control..

[3] Papaelias M., Roberts C., Davis C.L. (2008). A review on non-destructive evaluation of rails: State-of-the-art and future development. Proc. Inst. Mech. Eng. Part F J. Rail Rapid Transit.

[4] Gao P., Wang C., Zhi Y., Li Y. (2014). Defect classification using phase lag information of EC-GMR output. Nondestruct. Test. Eval..

[5] Nabavi M.R., Nihtianov S.N. (2012). Design Strategies for Eddy-Current Displacement Sensor Systems: Review and Recommendations. IEEE Sens. J..

[6] Fleming A.J. (2013). A review of nanometer resolution position sensors: Operation and performance. Sens. Actuators A Phys..

[7] Fahmi A., Kashyzadeh K.R., Ghorbani S. (2022). A comprehensive review on mechanical failures cause vibration in the gas turbine of combined cycle power plants. Eng. Fail. Anal..

[8] Lu M., Xie Y., Zhu W., Peyton A., Yin W. (2019). Determination of the Magnetic Permeability, Electrical Conductivity, and Thickness of Ferrite Metallic Plates Using a Multifrequency Electromagnetic Sensing System. IEEE Trans. Ind. Inform..

[9] Yin W., Peyton A.J. (2007). Thickness measurement of non-magnetic plates using multi-frequency eddy current sensors. NDT E Int..

[10] Rosado L.S., Gonzalez J.C., Santos T.G., Ramos P.M., Piedade M. (2013). Geometric optimization of a differential planar eddy currents probe for non-destructive testing. Sens. Actuators A Phys..

[11] Chen G.L., Zhang W.M., Zhang Z.J., Jin X., Pang W.H. (2018). A new rosette-like eddy current array sensor with high sensitivity for fatigue defect around bolt hole in SHM. Ndt E Int..

[12] She S.B., Liu Y.Z., Zhang S.J., Wen Y.Z., Zhou Z.J., Liu X.K., Sui Z.H., Ren D.T., Zhang F., He Y.Z. (2021). Flexible Differential Butterfly-Shape Eddy Current Array Sensor for Defect Detection of Screw Thread. IEEE Sens. J..

[13] Chen T., He Y., Du J. (2018). A High-Sensitivity Flexible Eddy Current Array Sensor for Crack Monitoring of Welded Structures under Varying Environment. Sensors.

[14] Zhang H.Y., Ma L.Y., Xie F.Q. (2019). A method of steel ball surface quality inspection based on flexible arrayed eddy current sensor. Measurement.

[15] Lu M.Y., Meng X.B., Huang R.C., Chen L.M., Peyton A., Yin W.L. (2021). Lift-off invariant inductance of steels in multi-frequency eddy-current testing. Ndt E Int..

[16] Ona D.I., Tian G.Y., Sutthaweekul R., Naqvi S.M. (2019). Design and optimisation of mutual inductance based pulsed eddy current probe. Measurement.

[17] Wang H., Feng Z. (2013). Ultrastable and highly sensitive eddy current displacement sensor using self-temperature compensation. Sens. Actuators A Phys..

[18] Wang H., Ju B., Li W., Feng Z. (2014). Ultrastable eddy current displacement sensor working in harsh temperature environments with comprehensive self-temperature compensation. Sens. Actuators A Phys..

[19] Mizuno T., Enoki S., Asahina T., Suzuki T., Maeda H., Asahi T., Shinagawa H. (2008). Output voltage characteristics of eddy current displacement sensor for various heat treatments of measuring objects. Trans. Inst. Electr. Eng. Jpn. A.

[20] Yating Y., Tuo Y., Pingan D. (2012). A new eddy current displacement measuring instrument independent of sample electromagnetic properties. NDT E Int..

[21] Li Y., Sheng X., Lian M., Wang Y. (2015). Influence of tilt angle on eddy current displacement measurement. Nondestruct. Test. Eval..

[22] Walton J.F., Heshmat H., Tomaszewski M.J. (2008). Testing of a small turbocharger/turbojet sized simulator rotor supported on foil bearings. J. Eng. Gas. Turb. Power.

[23] Mizumoto H., Arii S., Yabuta Y., Tazoe Y. Vibration Control of a High-Speed Air-Bearing Spindle using an Active Aerodynamic Bearing. Proceedings of the International Conference on Control, Automation and Systems (Iccas 2010).

[24] Ding T., Chen X. (2006). Eddy current sensor coil for measuring gaps between curved surfaces. J. Tsinghua Univ. Sci. Technol..

[25] Hu Y., Ding T., Wang P. (2013). Characteristics of eddy current effects in curved flexible coils. J. Tsinghua Univ. Sci. Technol..

[26] Zhang Y.-H., Sun H.-X., Luo F.-L. (2009). Suppression method of a probe-coil’s lift-off noise for the curved specimen with small curvature radius in eddy-current testing. Proc. CSEE.

[27] Zhan H., Wang L., Wang T., Yu J. (2020). The Influence and Compensation Method of Eccentricity for Cylindrical Specimens in Eddy Current Displacement Measurement. Sensors.

[28] Sheng X.J., Li Y., Lian M., Xu C., Wang Y.Q. (2016). Influence of Coupling Interference on Arrayed Eddy Current Displacement Measurement. Mater. Eval..

[29] Chiba A., Chiba A., Fukao T., Ichikawa O., Oshima M., Takemoto M., Dorrell D.G. (2005). 18—Displacement sensors and sensorless operation. Magnetic Bearings and Bearingless Drives.

[30] Pawlenka T., Skuta J. Shaft Displacement Measurement Using Capacitive Sensors. Proceedings of the 2021 22nd International Carpathian Control Conference (ICCC).

[31] Li H.W., Liang Z.Q., Pei J.J., Jiao L., Xie L.J., Wang X.B. (2018). New Measurement Method for Spline Shaft Rolling Performance Evaluation using Laser Displacement Sensor. Chin. J. Mech. Eng..

[32] Reda K., Yan Y. (2019). Vibration Measurement of an Unbalanced Metallic Shaft Using Electrostatic Sensors. IEEE Trans. Instrum. Meas..

[33] Liu F.X., Yang Y., Jiang D., Ruan X.B., Chen X.L. (2017). Modeling and Optimization of Magnetically Coupled Resonant Wireless Power Transfer System With Varying Spatial Scales. IEEE Trans. Power Electron..

[34] Raju S., Wu R.X., Chan M.S., Yue C.P. (2014). Modeling of Mutual Coupling Between Planar Inductors in Wireless Power Applications. Ieee Trans. Power Electron..

[35] Yating Y., Pingan D. (2008). Two approaches to coil impedance calculation of eddy current sensor. Proc. Inst. Mech. Eng. Part C J. Mech. Eng. Sci..

[36] Li Y.M., Wu J., Guo Q. (2020). Electromagnetic Sensor for Detecting Wear Debris in Lubricating Oil. IEEE Trans. Instrum. Meas..

[37] Yu Y.T., Du P.G. Optimization of an eddy current sensor using finite element method. Proceedings of the IEEE International Conference on Mechatronics and Automation.

[38] Yu Y.T., Du P.A., Wang Z.W. Study on the electromagnetic properties of eddy current sensor. Proceedings of the 2005 IEEE International Conference on Mechatronics and Automations.

[39] Welsby S.D., Hitz T. (1997). True position measurement with eddy current technology. Sensors.

[40] Cao F., Wang S.R., Wang G.Q., Li Y.D. The dynamics simulation analysis on turbocharger turbine-shaft system. Proceedings of the International Conference on Mechanics and Architectural Design (MAD).

[41] Jinwei W., Zheng W., Xiujuan W., Hong H., Li Z., Haiyun P. Research on the connection technology of TiAl alloy turbine in diesel engine turbocharger. Proceedings of the 2014 ISFMFE—6th International Symposium on Fluid Machinery and Fluid Engineering.

[42] Wang W., Li X., Zeng Q., Lou F., Lai T., Hou Y. (2017). Stability analysis for fully hydrodynamic gas-lubricated protuberant foil bearings in high speed turbomachinery. J. Xi’an Jiaotong Univ..

